# Automatic Coregistration Between Coronary Angiography and Intravascular Optical Coherence Tomography: Feasibility and Accuracy

**DOI:** 10.1016/j.jacasi.2021.07.002

**Published:** 2021-09-21

**Authors:** Hui Qin, Chunming Li, Yingguang Li, Jiayue Huang, Fan Yang, Takashi Kubo, Takashi Akasaka, Changyan Xiao, Juan Luis Gutiérrez-Chico, Shengxian Tu

**Affiliations:** a College of Electrical and Information Engineering, Hunan University, Changsha, China; b Biomedical Instrument Institute, School of Biomedical Engineering, Shanghai Jiao Tong University, Shanghai, China; c Kunshan Industrial Technology Research Institute Co Ltd, Kunshan, China; d Department of Cardiovascular Medicine, Wakayama Medical University, Wakayama, Japan; e Cardiology Department, Ruijin Hospital, Shanghai Jiao Tong University School of Medicine, Shanghai, China

**Keywords:** angiography, image registration, optical coherence tomography, percutaneous coronary intervention, CAG, coronary angiography, IQR, interquartile range, IVUS, intravascular ultrasound, LAD, left anterior descending coronary artery, OCT, optical coherence tomography, SB, side branch

## Abstract

This study sought to evaluate a novel approach for automatic coregistration of optical coherence tomography (OCT) and coronary angiography. Lumen diameters and side branches from both coronary angiography and OCT were used to create 2 feature sets. Subsequently, a 2-step coregistration approach was performed on the feature sets for matching of each OCT cross section on the angiographic centerline. For validation, all side branches with ≥1.0 mm diameter were identified and used as paired fiduciary landmarks. Geographical error was defined as the distance between the automatically coregistered and the true-paired landmarks. Altogether 212 vessels from 181 patients were analyzed. Mismatch of coronary angiography and OCT occurred in 64 of 1,530 reference landmarks. Median geographical error was 0.32 (interquartile range: 0.00-0.56) mm. The mean time for coregistration was 20.69 ± 1.07 seconds. In conclusion, fast and automatic coregistration of OCT and angiography using a single standard angiographic loop is feasible and accurate.

Intracoronary optical coherence tomography (OCT) allows detailed assessment of luminal dimension, plaque morphology, and interventional devices, thus offering a clear incremental value for percutaneous coronary intervention guidance in specific patient subsets (1). Automatic coregistration of OCT and coronary angiography (CAG) is useful to understand the anatomic correspondence between both imaging techniques, thus being instrumental for optimal stent sizing and positioning to select the optimal landing zones, an approach that can prevent ulterior procedural and long-term complications.

In the present study, we developed a novel method for automatic OCT-CAG coregistration that can be easily implemented with minimal requirements: a single standard angiographic projection and the OCT pullback. This approach circumvents the need for additional dedicated acquisitions and hence does not alter the ordinary diagnostic workflow in the catheterization laboratory. The feasibility and accuracy of this novel approach in unselected patients are unknown hitherto.

## Methods

### Study design and materials

All patients with CAG and OCT imaging data from a recent study (2) were included for post hoc validation of the coregistration approach. Analysis of imaging data was performed at an academic core laboratory (CardHemo, Med-X Research Institute). The patient population and details for the acquisition of CAG and OCT images have been reported elsewhere (2). The study complied with the Declaration of Helsinki for investigation in human beings, and the analysis was approved by the Institutional Review Board of Wakayama Medical University. All patients provided informed consent for enrollment in the institutional database for potential future investigations.

### Coregistration between x-ray angiography and OCT

The lumen contour of the interrogated vessel and its side branches (SBs) were automatically delineated on both angiography and OCT, using 2 software packages (AngioPlus Core and OctPlus, Pulse Medical Imaging Technology) (2,3). Subsequently, lumen diameters and relative distance between SBs were used to construct the corresponding feature sets for angiography and OCT. Finally, a 2-step coregistration approach, consisting of coarse and precise coregistration, was performed on the feature sets to determine the position of each OCT cross section on the angiographic centerline. The coarse coregistration relied on the sliding window method. In light of the limited length of the OCT pull back, the OCT feature set was used as a window to slide over the angiography feature set. A match score was computed based on the difference between the 2 feature sets within the sliding window. By maximizing the match score, the coarse registration was achieved with the assumption that OCT cross sections were evenly distributed along the angiographic centerline. The precise coregistration, applied a nonrigid point matching algorithm on top of the coarse coregistration to further align the relative position of each OCT cross section with respect to the angiographic centerline. The output guiding the precise coregistration was the optimal correspondence between each OCT image frame and the angiographic centerline point.

### Evaluation of coregistration error

All SBs with ≥1.0 mm diameter in the interrogated vessel were identified on OCT and angiography at the core lab by an experienced analyst and used as the ground truth to evaluate the proposed coregistration method. To avoid bias in selection of the ground truth, the ground truth was prepared before evaluation of the coregistration method. The OCT cross sections at the bifurcation carinas were paired with the corresponding angiographic carinas and used as fiduciary landmarks. Geographic error was defined as the distance between the automatically coregistered landmark position and the true position of the paired landmark on angiography. For each paired landmark, coregistration mismatch was defined as a geographic error ≥1.0 mm. Accurate coregistration was defined as a geographic error <1.0 mm. Vessel-level mean geographic error <1.0 mm was considered a successful coregistration.

### Statistical analysis

Statistical assessments were performed with MedCalc (version 14.12; MedCalc Software). Quantitative variables were presented as mean ± SD or as median (interquartile range [IQR]) as appropriate. Categorical variables were presented as counts (percentages). Continuous variables were compared with unpaired Student's *t*-test or Mann-Whitney *U* test, as appropriate, whereas categorical variables were compared with Fisher exact test. One-way analysis of variance test was used to compare continuous variables throughout categorical variables with >2 categories. The influence of some vessel parameters on geographic error was explored with Pearson correlation test and linear regression. The “n – 1” chi-square test was used for the comparison between accuracy. A 2-sided value of *P ≤* 0.05 was considered to be statistically significant.

## Results

Patient and vessel characteristics have been previously reported (2). Briefly, 212 coronary arteries from 181 patients were analyzed. The left anterior descending coronary artery (LAD) was the most frequently imaged vessel (n = 128, 60.4%), followed by the right coronary artery (n = 46, 21.7%). Complex coronary lesions were well represented in the sample: 97 bifurcation lesions (45.8%), 47 sequential lesions (22.2%), and 80 vessels with diffuse coronary disease (37.7%) (2).

**Figure 1:**
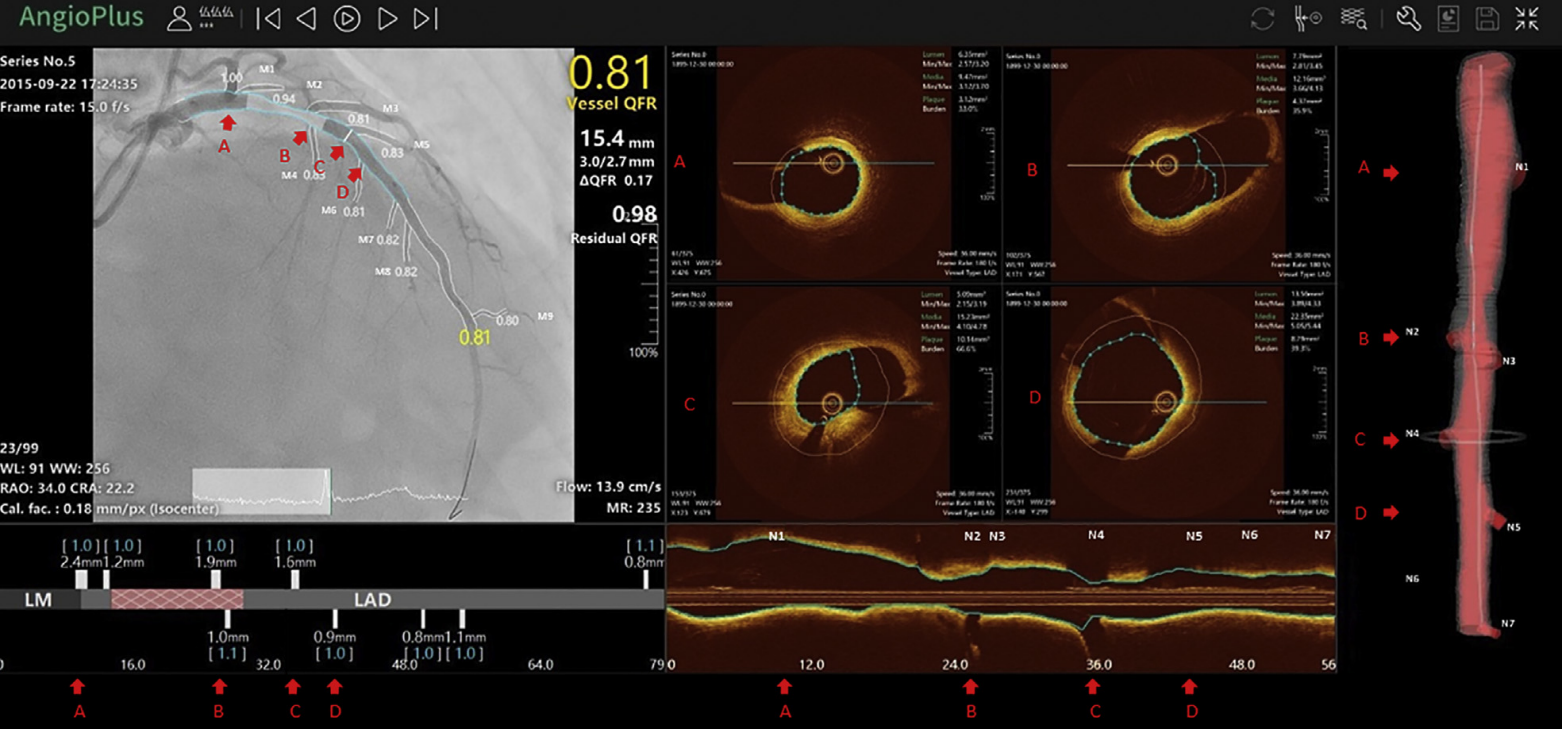
Representative Example of Coregistration Between Angiography and OCT M1 to M9 and N1 to N7 were the side branches detected on angiography and optical coherence tomography (OCT) images, respectively. Six pairs of side branches were accurately matched, where M1, M3, M4, M5, M6, and M7 correspond to N1, N2, N3, N4, N5, and N7, respectively. The marker positions **(A to D)** at angiographic centerline and OCT longitudinal view and 3-dimensional view **(red arrows)** were synchronized after coregistration.

Coregistration between x-ray angiography and OCT could be completed in all 212 vessels (feasibility 100%). Figure 1 shows a representative example. After coregistration, each OCT image frame was linked to the angiographic centerline point.

### Geographic error and accuracy

A total of 1,530 paired landmarks from 212 vessels were manually identified to evaluate the coregistration accuracy. The difference in the number of SBs detected by angiography versus OCT ranged from 0.00 to 6.00, with a median value of 1.00 (IQR: 1.00-2.00; *P <* 0.001). Coregistration mismatch occurred in 64 of 1,530 paired landmarks and in 4 of 212 vessels. Thus, accurate coregistration was achieved in 1,466 of 1,530 landmarks (96%) and in 208 of 212 vessels (98%). Geographic error ranged from 0.00 to 2.85 mm, with a median value of 0.32 mm (IQR: 0.00-0.56 mm; *P <* 0.001). Incorporation of precise coregistration on top of coarse coregistration significantly improved the accuracy (63% vs 96%; *P* < 0.001). Table 1 shows the comparison of coarse and precise coregistration performance in different vessels. Mean geographic error per vessel showed negligible correlation with mean lumen diameter (*r* = 0.13; *P =* 0.068) or vessel length (*r* = −0.15; *P =* 0.031), as assessed by OCT.

**Table 1:** Comparison of Coarse and Precise Coregistration Performance in Different Vessels

Vessel	Total Landmarks	Coarse Coregistration		Precise Coregistration	
		Geographic Error (mm)	Accuracy (%)	Geographic Error (mm)	Accuracy (%)
LAD	978	0.65 (0.20-1.26)	66.56	0.31 (0.00-0.53)	97.24
LCx	270	1.11 (0.38-1.94)	47.41	0.20 (0.00-0.56)	94.81
RCA	282	0.60 (0.19-1.54)	65.60	0.36 (0.18-0.70)	91.84

Values are mean (interquartile range) unless otherwise indicated.

LAD = left anterior descending coronary artery; LCx = left circumflex artery; RCA = right coronary artery.

### Subgroup analysis

The accuracy of coregistration was 97%, 95%, and 92% per landmark and 99%, 97%, and 96% per vessel for LAD, left circumflex, and right coronary artery, respectively. Geographic error varied significantly among different vessels: mean: 0.31 mm (IQR: 0.00-0.53 mm) in LAD, 0.36 mm (IQR: 0.18-0.70 mm) in right coronary artery, 0.20 mm (IQR: 0.00-0.56 mm) in left circumflex (*P =* 0.022 for all).

Geographic error was significantly larger in vessels with <4 SBs than in vessels with ≥4 SBs (mean: 0.36 mm [IQR: 0.17-0.73 mm] vs 0.21 mm [IQR: 0.00-0.55 mm]; *P =* 0.007).

### Computational performance

The average coregistration time was 20.69 ± 1.07 seconds using an off-the-shelf computer with hexacore Intel i7-9750 processor (2.60 GHz; Intel Corporation) and 16 GB of RAM.

## Discussion

The main findings of the present study can be summarized as follows: 1) automatic OCT-CAG coregistration is feasible and accurate in an unselected real-world series of patients, using a single angiographic projection and circumventing the need for simultaneous acquisition; 2) precise coregistration based on a nonrigid point-matching algorithm significantly improves the coregistration performance; 3) the hereby proposed method for automatic OCT-CAG coregistration is fast enough to be implemented into routine clinical practice.

To the best of our knowledge, this is the first study to present and validate a completely automatic method for real-time coregistration of OCT and CAG, only requiring a single angiographic projection and without need of simultaneous filming of the optical catheter during acquisition of the angiography.

Several methods (4, 5, 6) have been recently proposed to coregister angiography and intracoronary imaging. Most of these methods require 3-dimensional reconstruction of OCT/intravascular ultrasound (IVUS) images (4,5) or simultaneous filming of the OCT/IVUS transducer and angiography during the whole image acquisition (6), thus adding complexity and accessory steps to the basic diagnostic workflow, eventually resulting in an increased radiation dose for the patient.

Conversely, the novel method proposed in this study does not require the detection of exactly the same number of SBs in both angiography and OCT and can still automatically coregister both images with high accuracy, in a timely manner, and without altering the basic diagnostic workflow in the catheterization laboratory. Using the take-off points of SBs as fiduciary landmarks and a nonrigid point-matching algorithm resulted in accurate coregistration of 96% of the landmarks and 98% of the scanned vessels, with a median geographic error of 0.32 mm. A key feature for the optimal performance of the method is the 2-step matching approach, incorporating the precise coregistration step that significantly improved the accuracy without a relevant increase of the computational time. This is particularly important because of the following factors. 1) The angiography might have more foreshortening in curved segments than in straight segments in suboptimal angiography projections. Assumption of even distribution of the OCT frames between consecutive SB landmarks will be inaccurate if the segment is curved. 2) The currently available OCT imaging systems are inevitably associated with shortening/elongation distortion of vascular structures caused by cardiac motion artifact (7). Thus, the OCT frames were not evenly distributed along the vascular centerline, which can be overcome by using nonrigid coregistration. The coregistration approach still worked even though the numbers of SBs were not identical in angiography and in OCT. However, the number of SBs seems to be more relevant for an accurate coregistration: the best performance was observed in vessels with 4 or more SBs. This possibly explains why the highest coregistration accuracy was observed on the LAD, which is the coronary artery that usually has more SBs to guide the matching.

Novel systems such as the OPTIS Integrated System from Abbott have been recently developed, allowing real-time coregistration between OCT and angiography. However, these systems are based on simultaneous filming of the OCT probe in angiography during the whole image acquisition, thus resulting in a small increase of the radiation dose for the patients. Furthermore, for technical reasons, it can fail in some challenging cases and is not currently available in many catheterization laboratories worldwide. Conversely, the hereby proposed method provides rapid coregistration, without altering the basic diagnostic workflow in the catheterization laboratory. More importantly, this novel method can be retrospectively applied to any OCT pull back, provided an angiographic view of the same vessel is amenable.

## Conclusions

Automatic coregistration of OCT and angiography is feasible without altering the basic diagnostic workflow in the catheterization laboratory with additional acquisitions. The high accuracy and fast coregistration time of this approach enables its implementation in routine clinical practice.

## Funding Support and Author Disclosures

This study is supported by the National Key Research and Development Program of China and the Natural Science Foundation of China (grants 81871460 and 82020108015). Dr Tu has received research support from Pulse Medical Imaging Technology. All other authors have reported that they have no relationships relevant to the contents of this paper to disclose.
